# Evaluating the research parameters available on the Sysmex^®^ XN-series hematology analyzers as markers of dysplasia in peripheral blood

**DOI:** 10.1515/almed-2025-0003

**Published:** 2025-02-04

**Authors:** Vicente Aguadero, María López, Míriam Ruíz, Diana Regidor, Gemma Celma

**Affiliations:** Laboratory of Sant Joan Despí Moisès Broggi Hospital, Consorci del Laboratori Intercomarcal de l’Alt Penedès, l’Anoia i el Garraf (CLILAB Diagnòstics), Vilafranca del Penedès, Barcelona, Spain; Laboratoy of Vilafranca Del Penedès, 504109 Consorci del Laboratori Intercomarcal de l’Alt Penedès, l’Anoia i el Garraf (CLILAB Diagnòstics), Vilafranca del Penedès, Barcelona, Spain

**Keywords:** dysplasia, neutrophil granularity index (neu-Gi), platelet distribution width (PDW), immature platelet fraction (IPF), Sysmex

## Abstract

**Objectives:**

Myelodysplastic syndromes (MDS) are clonal hematopoietic disorders characterized by peripheral blood cytopenias, cellular dysplasia and risk for progression into acute leukemia. Recent studies reveal that some research parameters available on Sysmex XN-1000^®^ hematology analyzers, including immature platelet fraction (IPF), Neutrophil Granularity Index (Neu-GI), or platelet distribution width (PDW), show a relationship with dysplasia in peripheral blood. The objective of this study was to examine the association between classic and research blood count parameters and the presence of dysplasia. The secondary objective was to develop a multivariate model that allows the prediction of dysplasia with high probability.

**Methods:**

Seventy-five patients older than 60 years with anemia, leukopenia or thrombocytopenia, without vitamin B12 and folate deficiency or hematological diseases underwent testing with the Sysmex XN-1000 analyzer.

**Results:**

Dysplasia was confirmed in 32 % of patients, with significant differences in Neu-GI, PDW and IPF count between the groups of patients with and without dysplasia. Neu-GI was the parameter with the highest predictive value (AUC=0.98), with such value not increasing significantly after the addition of PDW or PIF. A Neu-GI value≤146ch predicts dysplasia with a positive predictive value=90 %.

**Conclusions:**

Neu-GI is the parameter most strongly associated with dysplasia. A Neu-GI value≤146ch indicates a high probability of dysplasia and supports indication for a blood smear review. Additionally, values>152ch indicate a low probability of dysplasia.

## Introduction

Myelodysplastic syndromes (MDS) represent a heterogeneous group of clonal hematopoiesis disorders. These diseases are characterized by different levels of cytopenias in peripheral blood, distinctive morphological abnormalities in hematopoietic elements (dysplastic changes), and an increased risk for progression into acute myeloblastic leukemia [1], [2], [3].

Screening for MDS should be performed in the presence of normo-macrocytic anemia without vitamin B12 and folate deficiency or hepatic abnormalities, and with associated leukopenia or thrombocytopenia [4].

The first step for diagnosis includes a basic blood count test and a peripheral blood smear test examined under the microscope to detect potential dysplastic changes [4]. Apart from the classic blood count parameters, new parameters are available on Sysmex XN-Series autoanalyzers, some of which are still investigational. These parameters include analytes potentially associated with dysplasia in peripheral blood [5]. Evidence has been provided of an association between parameters such as neutrophil granulatory index (Neu-GI) or immature platelet fraction (IPF) and the presence of dysplasia. Hence, these parameters emerge as potential markers for early diagnosis of MDS [6], 7]. However, evidence is limited and the studies available consider these parameters separately. This study included all classic and investigational blood count parameters to identify the combination that best predicts the presence of dysplasia in peripheral blood.

### Objectives

Determining the level of association between classic and investigational parameters available on Sysmex XN autoanalyzers, both, separately and in combination, and the presence of dysplasia.

Developing a formula, index or cut-off value to predict or exclude with a high probability the presence of dysplasia to help determine whether a blood smear test is indicated or not.

## Materials and methods

For a period of six months, we included patients undergoing a routine blood count on the XN-1000 (Sysmex Corporation, Kobe, Japan) hematology analyzer, who met the following criteria: (i) age>60 years; (ii) normocytic or macrocytic anemia + leukopenia or thrombocytopenia; (iii) no folate/vitamin B12 deficiency; no liver disease; no anti-cancer therapy; and no previous history of hematologic disease. Data collected from each patient included age, sex, type of cytopenia (anemia + thrombocytopenia/anemia + leukopenia/pancytopenia), type of anemia (normocytic/macrocytic), added to results for classic blood count parameters (hemoglobin, leukocyte count, erythrocyte count, platelet count, neutrophil-to-monocyte ratio, mean corpuscular volume (MCV), and leukocyte distribution width), and results for investigational parameters (IPF, Neu-GI, platelet distribution width (PDW), macrocyte percentage (Macro-R), microcyte percentage (Micro-R), immature reticulocyte fraction (IRF)), and finding of dysplasia (YES/NO) in any of the three blood cell series in the peripheral blood smear test. Criteria of dysplasia [4], 8]:

– Dyserythropoiesis: abnormal erythroblast maturation and binucleation; erythrocytes with erythrocyte inclusions (Cabot rings, Howell-Jolly bodies and basophilic stippling).
– Dysgranulopoiesis: hypogranularity, hypersegmentation, hyposegmentation (Pseudo-Pelguer), aberrant segmentation, myelokathexis and Döhle Body inclusions.
– Dysmegakaryopoiesis: giant, degranulated platelets, abnormal distribution of granularity or presence of pseudopodes.

Blood smears were reviewed by two experienced observers. Pearson’s chi-squared test (χ^2^) was used to demonstrate the absence of significant differences in the assessments. Patients were categorized into two groups according to blood smear results: finding of dysplasia/absence of dysplasia. Normal distribution of quantitative variables was assessed using the Kolmogorov-Smirnoff test. For quantitative variables, differences in means between the two groups were assessed using Student’s t-test or Mann-Whitney U test. For qualitative variables, differences in percentages were assessed using χ^2^. Significant variables were included in multivariate logistic regression analysis. By the backwards methods, variables that were not essential for the algorithm were iteratively removed and only the variables with the highest predictive value (estimated by the Wald Statistic value) for dysplasia were maintained. Outliers were detected by evaluation of Cook’s distance. The reliability of regression models was assessed by the area under the curve (AUC) and the percentage of accuracy. Delong test was used for comparison of AUCs from the different regression models. Cut-off values were established from coordinate points on the ROC curves that represent the desired sensitivity and/or specificity. Statistical analyses were performed using R Commander 3.6.1 and SPSS version 25.0.

## Results

A total of 75 patients were included in the study. Dysplasia was confirmed in 24 cases (32 %). The mean or number of cases in each group for each variable is shown in Table 1.

**Table 1:** Number of cases and means for the different blood cell count parameters examined, according to the finding or not of dysplasia in peripheral blood smear.

	Dysplasia		Total	Sig.
	No (cases/mean)	Yes (cases/mean)		
Sex				
Male	34	16	50	0.361^a^
Female	18	7	25	
Total	49	21	75	
Cytopenia				
Anemia + Leukopenia	2	1	3	0.337^a^
Anemia + Thrombocytopenia	21	14	35	
Pancytopenia	28	9	37	
Total	51	24	75	
Anemia				
Normocytic	17	10	27	0.517^a^
Macrocytic	34	14	48	
Total	51	24	75	
Age, years	76	78		0.103^b^
Hemoglobin, g/L	96.1	96.2		0.987^c^
Mean corpuscular volume, FL	100.0	97.4		0.139^b^
Erythrocytes, 10^12^/L	2.90	3.01		0.214^b^
Leukocytes, 10^12^/L	4.75	7.72		0.120^c^
Platelets, 10^9^/L	65	77		0.286^c^
Erythrocyte distribution width, %	16.2	16.1		0.791^c^
Neutrophils, %	61.5	61.1		0.459^b^
Monocytes, %	11.1	10.9		0.987^c^
Platelet distribution width, FL	13.4	16.3		<0.001^b^
Immature platelets, %	6.4	11.9		<0.001^c^
Neu-GI (SI)	153	144		<0.001^b^
Macrocytes, %	10.7	8.1		0.299^c^
Microcytes, %	2.1	2.4		0.134^c^
Immature reticulocytes, %	20.0	20.7		0.378^b^
Neutrophils, %/ monocytes, %	6.94	7.8		0.728^c^

Sig.*,* level of significance (p<0.05 indicates statistical significance). ^a^Chi-squared test; ^b^Student’s t-test; ^c^Mann Whitney U test. SI, scatter intensity; FL, femtoliters.

No between-observer variability was found in the evaluation of blood smears (p<0.05, χ^2^).

**Figure 1:**
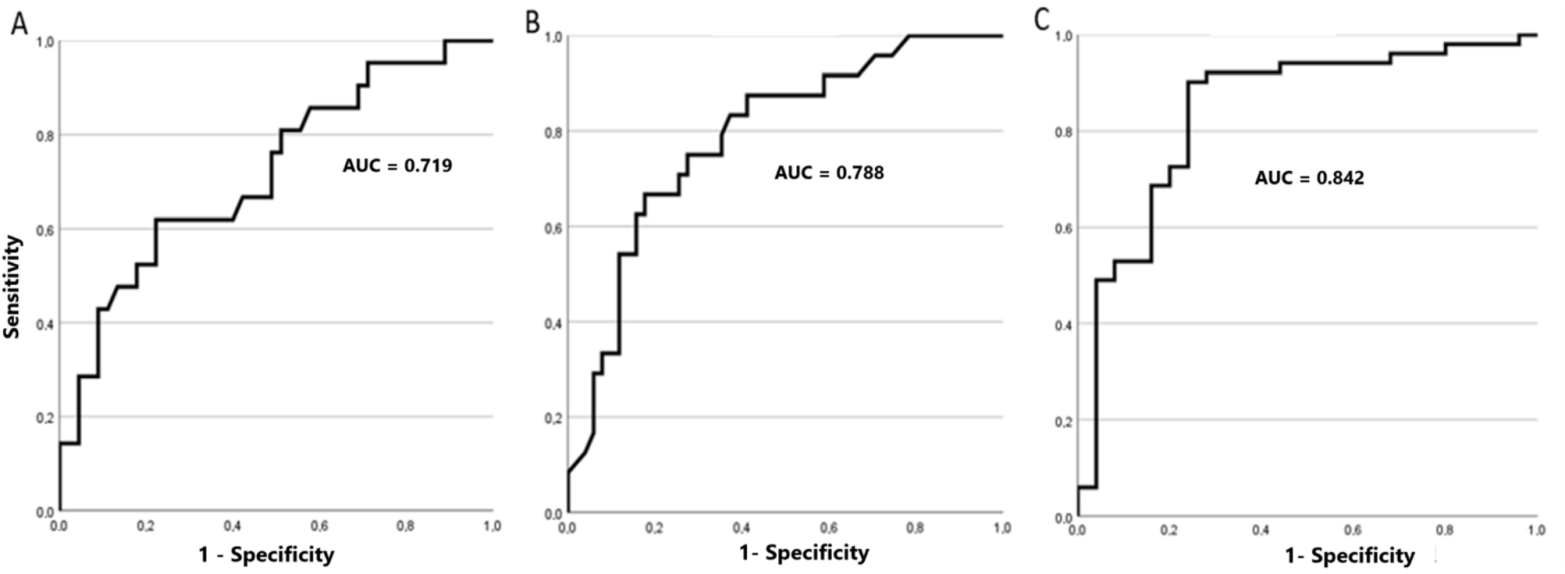
ROC curves for the parameters with a significant association with the presence of dysplastic changes in peripheral blood smear in bivariate analysis. (A) Platelet distribution width (PDW), (B) immature platelet fraction (IPF), (C) neutrophil granularity index (Neu-GI).

The bivariate study excluded a significant association between dysplasia and any type of anemia or cytopenia. Of the quantitative parameters studied, statistically significant differences were only observed in Neu-GI, PDW and IPF between the two groups (Table 1). The presence of dysplasia was associated with low values of Neu-GI and high values of PDW and IPF, with AUCs of 0.842, 0.788 and 0.842, respectively (Figure 1).

**Figure 2:**
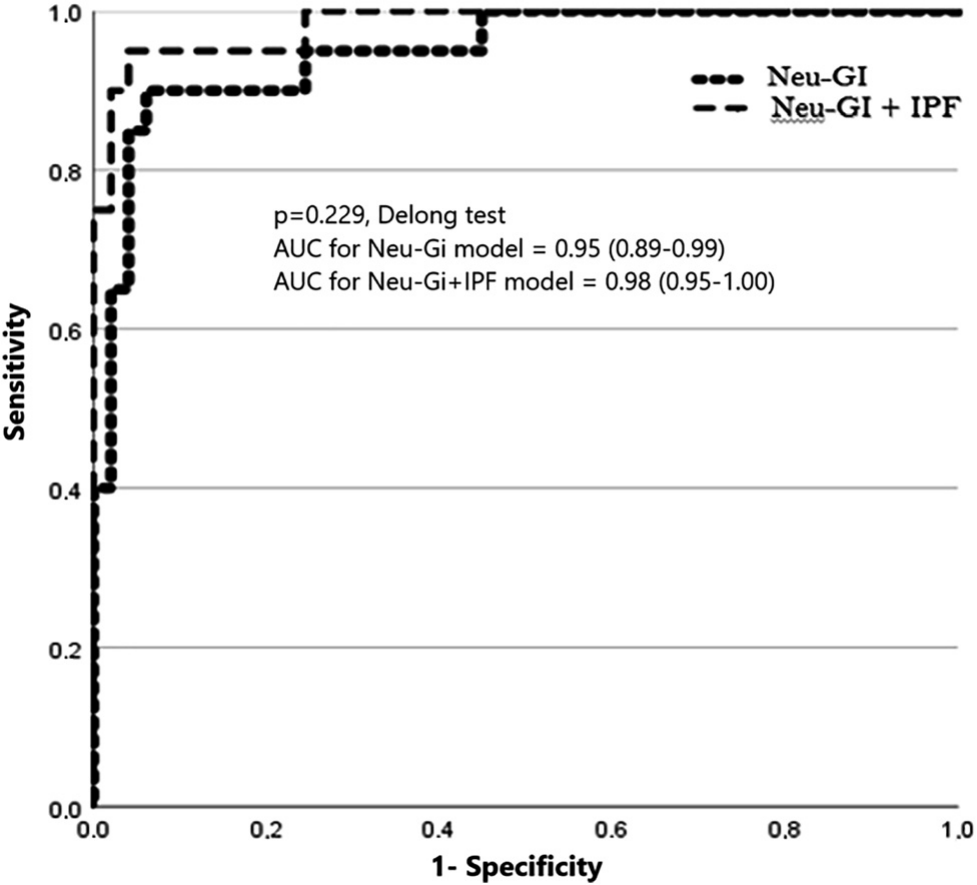
Comparison of the ROC curves of the dysplasia predictive model including Neu-GI and IPF, and the model solely including Neu-GI. Significant differences were established at p*<*0.05 (Delong test).

Multivariate analysis excluded a significant association between PDW and dysplasia (p=0.47, EW=0.5). Neu-GI was found to have the strongest association with dysplasia (p=0.001, EW=10.2), followed by IPF (p=0.030, EW=4.7). Two models were constructed, a model combining Neu-GI + IPF (AUC=0.95, E=94 %), and another model solely including Neu-GI (AUC=0.98, E=92 %), without significant differences in their predictive value for dysplasia (p=0.229, Delong test) (Figure 2). Taking the Neu-GI model as a reference, a cut-off value of ≤ 146 scatter intensity predicts the presence of dysplasia with a positive predictive value of 90 % (83 % sensitivity, 96 % specificity, and 92 % negative predictive value (NPV)). To reach a NPV of 100 %, the cut-off value needs to be increased to a scatter intensity of 152.

## Discussion

The Neu-GI value is estimated from the side-scattering index of the laser directed by the fluorescent flow cytometer onto the cell, which is captured by the SSC (side-scattered light) channel. SSC provides information about the internal structure of the cell. If the complexity of a cell increases upon a change in functionality (e.g. by a reactive increase in toxic granulation in an inflammatory process, or a reduction of granulation originating from a central formation defect), the position of the neutrophil cloud along the X axis in the scattergram is affected. The parameter Neu-GI, expressed in the SI unit (scatter intensity) changes accordingly [9].

Our results unveil that Neu-GI is the variable with the highest association with dysplasia. Technically, the Neu-GI is an evolution of the investigational parameters Neu-X and Neu-Y available on Sysmex XE series analyzers. These parameters have been demonstrated to detect 75 % of cases of MDS with neutrophil hypogranulation [10]. Although Neu-GI is a parameter of the white blood series, only 56 % of cases in our study had leukopenia. This increases the relevance of Neu-GI as a marker of dysplasia, even when the white blood cell series is normal.

Although macrocytic anemia is the most frequent type of anemia, neither % Macro-R or MCV showed a correlation with dysplasia. It is worth noting that evidence has been provided of an association between MCV and a final diagnosis of MDS, but not of a direct relationship with dysplastic changes in peripheral blood [11], 12].

Notably, 96 % of cases had thrombocytopenia, which could have strengthened the association of parameters of this series, such as PDW and IPF. As a result, the AUC for a finding of dysplasia is higher than the one reported in previous studies in relation to the diagnosis of MDS [13], 14].

Despite MDS being a central cord syndrome, surprisingly, low IRF values were not associated with dysplasia. In contrast and unexpectedly, elevated levels of IPF were associated with MDS, as previously reported in the literature. Within the setting of MDS patients, this finding could be explained by the fact that aberrantly high percentage rates of IPF most likely reflect the presence of dysmegakaryopoiesis, as opposed to an increase in megakaryocytic activity [15].

Our study included patients in whom a MDS would have been suspected in routine practice upon a finding of anemia with concurrent cytopenia. However, in a high proportion of cases, anemia is the only abnormality found in blood cell count at diagnosis, cases that are not represented in our study. Hence, red blood cell parameters, such as macro/micro-R, IRF, MCV or ADW could have shown a more relevant role as predictors of dysplasia.

Dysplastic changes are not pathognomonic of MDS as they are also found in other hematological (myeloproliferative syndromes) and non-hematological (vitamin B12 and folate deficiency, toxic agents, neoplasms or infections) disorders [1], 2], 8]. For this reason, it would have been interesting to determine which cases in the dysplasia group had a final diagnosis of MDS and establish whether any of the investigational parameters used would have had value for differential diagnosis. This hypothesis could not be tested due to the delay in performing complementary studies (e.g. cytometry, molecular biology and cytogenetics), added to the high number of patients lost to follow up due to their referral to other centers for a final diagnosis. Based on the association observed between Neu-GI, IPF and PDW and dysplastic changes, it is necessary to perform a study where final diagnosis is considered to determine the true value of these investigational parameters for differential diagnosis of MDS.

## Conclusions

Only PDW, IPF and Neu-GI showed a significant association with the presence of dysplasia. Neu-GI showed the strongest association with dysplasia, with the inclusion of PDW or IPF not increasing the predictive power of the model significantly. In patients meeting inclusion criteria, a Neu-GI value≤146 indicated a high probability of peripheral blood dysplasia, thereby supporting the indication of a blood smear test. A Neu-GI value>152 indicated a very low probability of dysplasia.
